# Error-related potentials during continuous feedback: using EEG to detect errors of different type and severity

**DOI:** 10.3389/fnhum.2015.00155

**Published:** 2015-03-26

**Authors:** Martin Spüler, Christian Niethammer

**Affiliations:** Computer Science Department, University of TübingenTübingen, Germany

**Keywords:** error-related potential (ErrP), error-related negativity (Ne/ERN), feedback related negativity (FRN), brain-computer interface (BCI), human-computer interaction, performance monitoring, asynchronous classification

## Abstract

When a person recognizes an error during a task, an error-related potential (ErrP) can be measured as response. It has been shown that ErrPs can be automatically detected in tasks with time-discrete feedback, which is widely applied in the field of Brain-Computer Interfaces (BCIs) for error correction or adaptation. However, there are only a few studies that concentrate on ErrPs during continuous feedback. With this study, we wanted to answer three different questions: (i) Can ErrPs be measured in electroencephalography (EEG) recordings during a task with continuous cursor control? (ii) Can ErrPs be classified using machine learning methods and is it possible to discriminate errors of different origins? (iii) Can we use EEG to detect the severity of an error? To answer these questions, we recorded EEG data from 10 subjects during a video game task and investigated two different types of error (execution error, due to inaccurate feedback; outcome error, due to not achieving the goal of an action). We analyzed the recorded data to show that during the same task, different kinds of error produce different ErrP waveforms and have a different spectral response. This allows us to detect and discriminate errors of different origin in an event-locked manner. By utilizing the error-related spectral response, we show that also a continuous, asynchronous detection of errors is possible. Although the detection of error severity based on EEG was one goal of this study, we did not find any significant influence of the severity on the EEG.

## 1. Introduction

If a person makes or perceives an error, an error-related potential can be detected in the electroencephalogram (EEG) due to the person recognizing that error (Falkenstein et al., 2000). Recently, ErrPs have gained interest for the use in Brain-Computer Interface (BCI) applications, which give the user the ability to communicate by means of brain activity only. That an ErrP can be detected when a BCI delivers erroneous feedback, has been shown in several publications (Ferrez and del R Millan, 2008; Chavarriaga et al., 2014) and it has further been shown that the detection of ErrPs can be utilized to correct errors (Schmidt et al., 2012; Spüler et al., 2012a) or improve adaptation of the BCI (Llera et al., 2011; Spüler et al., 2012b). So far, ErrPs have mainly been utilized in BCIs with discrete feedback, which is why we want to investigate the detection of ErrPs in a continuous task toward the utilization of ErrP detection in continuous BCI applications. Since the interest of this study is not specifically in one of the components of the ErrP or the neurophysiological interpretation, but the investigation of the error-related response in general with regards to its utilization in continuous BCI systems, we use the term error-related potential (ErrP). As the ErrP in BCI applications consists of multiple components, ErrP is the commonly used term (Chavarriaga et al., 2014) in the BCI literature and generally considered as an umbrella term, which comprises all components of the event-related potential that can be measured in response to an error.

Error-related potentials were first studied in choice reaction tasks (Falkenstein et al., 1990) and two components of the event-related potential were described that can be measured as consequence of an error. The first component is the error-related negativity (ERN or Ne) (Falkenstein et al., 1991; Gehring et al., 1993), which is a negative potential peaking 50–100 ms after an erroneous response. Depending on the task, an error-related positive potential, called error positivity (Pe), follows the ERN. The Pe can be further divided into a frontocentral and a centroparietal component. The frontocentral Pe, which seems to be related to the P3a, appears directly after the ERN, while the late Pe appears in the centroparietal region with a latency of 200–400 ms after the error and seems to be related to the P3b (Ullsperger et al., 2014). Regarding the meaning of these components, it seems that the negative components are mostly linked to error processing (Krigolson and Holroyd, 2007a) and reward prediction (Holroyd et al., 2003, 2009), while the positive component is likely associated with conscious error perception (Wessel et al., 2011).

Depending on the experimental task, different variants of error-related potentials can be measured. If an error is indicated by feedback, a feedback-related negativity (FRN) can be measured frontocentrally 200–300 ms after the feedback during a reinforcement learning task (Holroyd and Coles, 2002). The FRN seems to be related to or even is the same component as the N200 (Holroyd et al., 2008). Further, it was shown that an ERN can also be measured if a subject is observing another subject making an error (van Schie et al., 2004). Lastly, it was shown that an ErrP can be measured during human-computer interaction (Ferrez and Millán, 2005) and interaction with a BCI (Ferrez and del R Millan, 2008). In comparison with the previously mentioned error-related potentials, the interaction ErrP is more complex with a small positive peak around 200 ms after BCI feedback, a negative peak at 250 ms (likely related to FRN), a positive peak at 320 ms (likely related to Pe) and another broad negative peak at around 450 ms (N400); but these latencies can differ depending on the experimental paradigm (Iturrate et al., 2013).

While the majority of the studies used tasks with discrete feedback, Krigolson and Holroyd (2006) have examined errors in a continuous tracking task and have shown that an ErrP can be measured if the cursor does not respond during this continuous task (Krigolson and Holroyd, 2007a). With the aim of studying ErrPs toward their utilization in BCI, Kreilinger et al. (2012) investigated ErrPs during continuous arm movement and tried to classify ErrPs by mapping the continuous feedback to time-discrete feedback and additionally displaying the discrete feedback. That a discretization of the feedback is not needed, was shown by Milekovic et al. (2012) in a study using electrocorticography (ECoG) instead of EEG. They could show that an error-related response during continuous feedback can be observed in the ECoG signal and also be classified (Milekovic et al., 2013). Since the ErrP is not only visible in the time-domain, but there is evidence that there also is an error-related frequency modulation in EEG (Llera et al., 2011; Omedes et al., 2013, 2014), mainly in the theta frequency range (Cavanagh and Frank, 2014), the frequency spectrum could be used for ErrP detection when there is only continuous feedback. Further, Milekovic et al. (2012) used the terms “execution error” (if the interface delivers erroneous feedback) and “outcome error” (if a goal of an action is not achieved, i.e., the user is making an error) and showed that machine learning methods can be used to discriminate different error types based on ECoG recordings. While the degree of an error is another property that has been shown to be reflected in the strength of the error-related negativity (Falkenstein et al., 2000), it has not yet been investigated in the context of BCI applications. Based on the results by Milekovic et al., the study presented in this paper aims at answering three questions: (i) Can ErrPs be found in EEG during a cursor control task with continuous feedback? (ii) Can machine learning methods be used to detect and discriminate execution and outcome errors in EEG? (iii) Can the severity of an error be detected in EEG?

## 2. Methods

### 2.1. Task description

**Figure 1:**
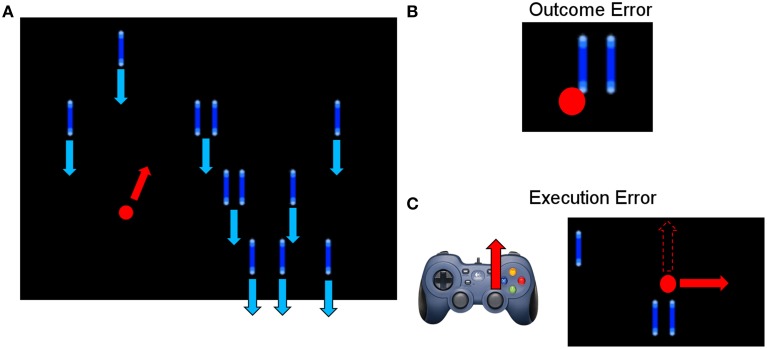
**(A)** Picture of the paradigm. The red and blue arrows indicate the movement direction of the objects (blue) and the cursor (red). The subject could move the red cursor with the gamepad to avoid a collision with one of the blue blocks, which were continuously falling down from the top of the screen. **(B)** Example of an outcome error, when the cursor collided with a block. **(C)** Example of an execution error, when the cursor moved for 2000 ms in a different direction than indicated by the subject through gamepad control. The dashed arrow in the screenshot indicates the expected movement direction, while the solid red arrow indicates the actual, erroneous direction.

The experimental task used in this study was similar to the one described by Milekovic et al. (2012), in which the subject had to play a simple video game (depicted in Figure 1). The subject used the right thumbstick of a gamepad to control the angle in which the cursor moved on the screen. The task was to avoid collisions of the cursor with blocks dropping from the top of the screen with a constant speed. The speed of the falling blocks was set to a level that the game was challenging and the player collided with a block from time to time. In case of a collision, the game continued for 1 s and then stopped. The delay of 1 s was introduced to make sure that the reaction measured in the EEG originates from the subject recognizing the collision (outcome error) and not from the game stopping or restarting. To study the execution error, which is happening when the interface delivers erroneous feedback, the angle of the cursor movement was modified for the duration of 2 s. The degree of modification was randomized (45°, 90°, 180° to either the left or the right side). The time between two execution errors was randomized to be between 5 and 8 s.

### 2.2. Experimental setup

10 healthy subjects (mean age: 24.1 ± 1.1 years) were recruited for this study. EEG was measured with two g.tec g.USBamp amplifiers and a Brainproducts Acticap System. 28 electrodes were placed on the scalp of the subject to measure EEG (at positions Fpz, AFz, F3, Fz, F4, F8, FC3, FCz, FC4, T7, C3, Cz, C4, T8, CP3, CPz, CP4, P7, P3, Pz, P4, P8, PO7, POz, PO8, O1, Oz, O2), while 3 electrodes were placed below the outer canthi of the eye and above the nasion for electrooculogram (EOG) recordings. The data was recorded with a sampling rate of 512 Hz and a 50 Hz notch filter was applied to filter out power line noise, as well as an additional bandpass filter between 0.5 and 60 Hz. The position of the thumbstick as well as information about outcome or execution errors was transmitted to the recording software using the parallel port of the computer.

### 2.3. Data analysis

The data was segmented into different trials with a length of 1 s: execution errors, time-locked to the start of an angle modification; outcome errors, time-locked to the collision event; and noError trials, when neither a collision nor an angle modification has happened during the trial or in the 1 s before or after the trial. For each subject, about 1 h of EEG was recorded, resulting on average in 597 ± 22 execution errors, 86 ± 30 outcome errors and 475 ± 39 noError trials. An EOG-based regression method (Schlögl et al., 2007) was used to reduce the effect of eye artifacts and the signal was re-referenced to the common average. For analysis of the power spectrum, we used the method by Welch (1967) on the time interval 0–1 s.

### 2.4. Event-locked classification

For the event-locked classification, we evaluated classification using three types of features: time domain features only, frequency domain features only, and the combination of both. To optimize the parameters for each feature set, we tested different parameters (e.g., regularization, time range) on data of subject S01 and finally used the parameters that worked best, which are stated below. With these parameters a cross-validation was performed on all 10 subjects to evaluate performance.

For the time domain features, we used the samples from all channels in the time range 0.2–0.9 s after an error event. For frequency domain features, we calculated the power spectrum using the method by Welch (1967) on the time range 0.2–0.9 s after an error event. The first 40 bins of the power spectrum for all channels were used as features for classification. For the combination of time domain and frequency domain features, we concatenated both feature vectors.

To estimate classification accuracies, we used a 10-fold cross-validation. As classifier, we used a Support Vector Machine (SVM) (Vapnik, 1998) using the LibSVM implementation (Chang and Lin, 2011) with a linear kernel and the hyperparameter set to the default value of *C* = 1. To investigate how well the error can be classified, outcome error and execution error, respectively, were classified against noError trials. We also classified execution errors against outcome errors, to see if the two types of errors can be discriminated. Since the number of trials was different for each class, the dataset was always balanced to obtain an even amount of trials for each class. To assess the significance of the results, we performed a permutation test with 1200 repetitions, in which for each repetition the vector containing the class labels was randomly permuted before training and the accuracy was calculated to obtain the significance level for *p* = 0.05.

To test if the subject's movements (due to gamepad control) or eye movements influence classification, classification was also done on the EOG data and on the recorded position of the thumbstick. The classification process itself was the same as for the EEG data, only the features were replaced by the time-domain EOG data and thumbstick position data, respectively.

One of the aims of this study was to investigate if it is possible to detect the severity of an error. Therefore, we tried to classify how well different angles of the execution error can be classified against each other. We additionally separated the execution error trials corresponding to the degree of deflection and performed a classification for each combination of two degrees. Since 45° and 315° is basically the same degree, but either to the left or to the right side, we also joined the trials for 45° and 315°, and 90° and 270°.

### 2.5. Asynchronous classification

Due to the missing information, when an error happens in online applications, an event-locked classification is not applicable online with continuous feedback. Therefore, we investigated how well error-related potentials can be classified asynchronously. For the asynchronous classification, a window with a length of 1 s was shifted over the whole signal by 62.5 ms steps. In each step, the window was classified whether or not it contained an error event. To ensure that training and testing data do not overlap, we performed a chronological 10-fold cross-validation in which EEG data was partitioned into 10 segments. The event-locked trials in 9 segments were used for training the classifier and then tested asynchronously on the remaining segment. This procedure resulted in an output every 62.5 ms, which labels each window as error or noError trial.

Since an asynchronous classification has higher time constraints to be able to run in real-time and we also found other parameters to yield better results in the asynchronous classification, we used different methods for signal processing and classification than for the event-locked analysis. For the final asynchronous classification, we used only spectral features because they performed superior to time-domain features or a combination of both features in the asynchronous classification. We used the maximum entropy method by Burg (1972) with a model order of 16 to estimate the power spectrum in the range of 1–12 Hz with a resolution of 1 Hz per bin. One of the most striking differences between the asynchronous and the event-locked classification is the time window used for spectral estimation and classification. The power spectrum was not estimated on the whole 1 s window, but only on a smaller time range. While we found the time range of 0.2–0.9 s to be optimal for the event-locked classification, we obtained best results for the asynchronous classification using a smaller time range of 0.1–0.5 s after an error event. While the reduction of the time range results in better classification performance, it also improves the reaction time of an asynchronous classification (having a delay of only 0.5 s instead of 0.9 s). Based on the features obtained after spectral estimation, we performed a feature selection based on *R*^2^-values (Spüler et al., 2011) to select the 20 best features. Those features were used to train a SVM (linear kernel, *C* = 1). Since the class imbalance is much higher for the outcome error, we used a weighted SVM in this case, which assigns different cost factors to the classes. We obtained best results when the cost factor C is 5 times higher for outcome errors than for noError trials. Based on the output of the SVM, a probabilistic output was assigned (Lin et al., 2007) and a weighted average of the last three probabilistic outputs was computed and taken as a final value. If this value was above a specified threshold, the current window was classified as an error.

## 3. Results

### 3.1. Neurophysiological analysis of error-related potentials

**Figure 2:**
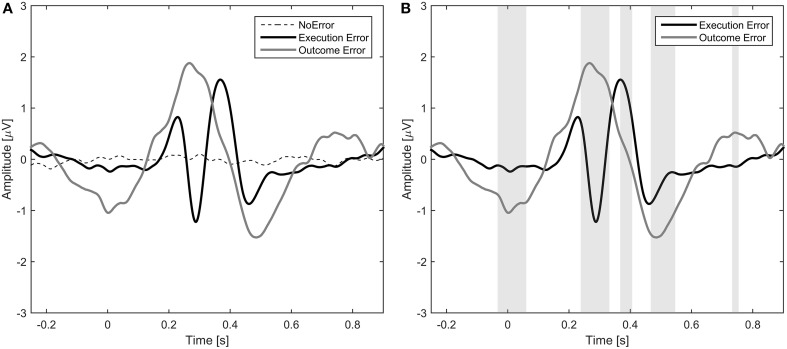
**(A)** Event-related potential at electrode FCz averaged over all subjects for NoError trials, execution errors and outcome errors. **(B)** Event-related potential for execution errors and outcome errors. The gray background denotes the time intervals with a significant difference between execution and outcome error (*p* < 0.05, Bonferroni corrected).

**Figure 3:**
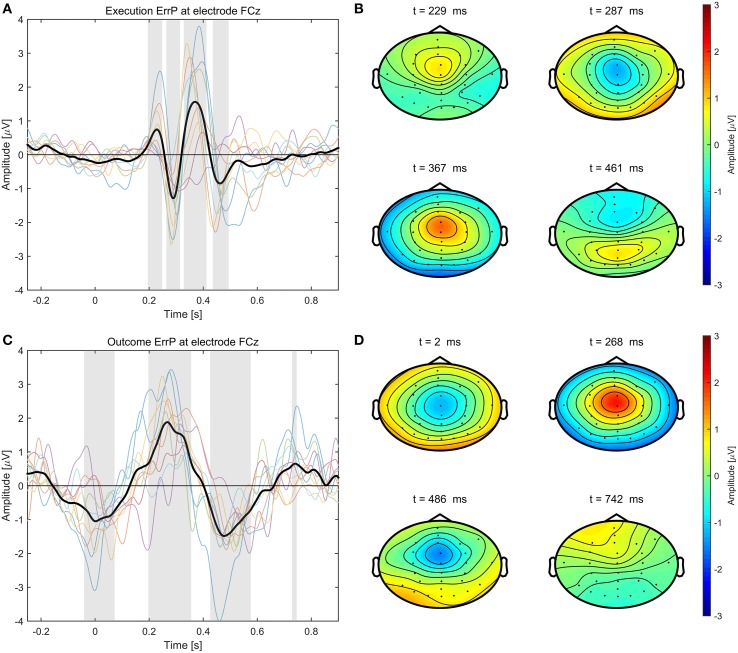
**Left:** Error-related potentials at electrode FCz for execution error **(A)** and outcome error **(C)**. For display of the ErrPs, the difference between the error trials and noError trials was calculated. The colored lines depict the ErrP for the different subjects, while the bold black line is the average over all subjects. Errors are happening at *t* = 0 ms. The gray background denotes the time intervals with a significant difference between error and noError trials (*p* < 0.05, Bonferroni corrected). **Right**: Scalp plots showing the topographic distribution of the error-related potential for execution **(B)** and outcome error **(D)** at the time of the maximum deflection for each of the significant time intervals.

The average event-related potentials for NoError trials, execution errors, and outcome errors are shown in Figure 2, along with the significant differences between execution and outcome error. Figure 3 shows the average difference waveform of execution error and outcome error at electrode FCz for all subjects, as well as the topographic distribution of the potential. It can be seen that a clear potential is visible for both kinds of error. The topographic distribution is similar for both errors and all subjects, with the maximum around electrode FCz and Cz. However, the waveform shape of the two error potentials differs strongly. For the execution error, we found a positive peak at 229 ms, a negative peak at 287 ms, a positive peak at 367 ms, and a small negative peak at 461 ms. In contrast, the outcome ErrP starts with a negative peak at 2 ms, followed by a positive peak at 268 ms, a negative peak at 486 ms, and a small positivity at 742 ms.

**Figure 4:**
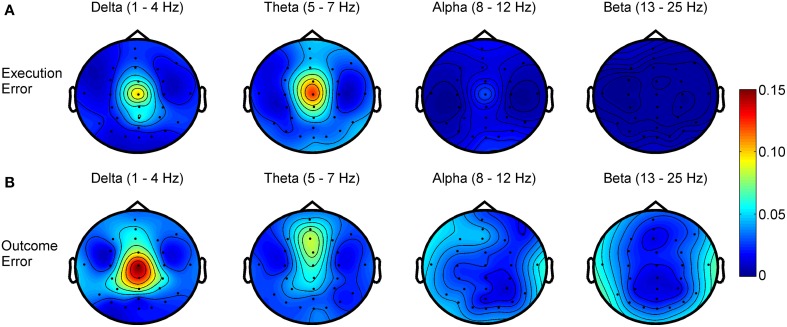
***R*^2^ values showing the difference in power for different frequency bands between noError trials and execution error (A) or outcome error (B), respectively**.

Regarding the frequency spectra of the observed error potentials (see Figure 4), we found activity mainly in the delta (1–4 Hz) and theta (5–7 Hz) frequency band for both errors, but the errors show a different spatial power distribution. For the execution error, the activity in both bands is strictly located at electrode Cz. For outcome errors, activity in the delta band can be seen mainly around Cz, while Fz and FCz show activity in the theta band.

By analyzing the execution error with regard to its severity, we did not find any significant effect (after correcting for multiple comparisons). Since the execution error does not differ depending on the degree, we did not show executions ErrPs separated by severity in this paper.

### 3.2. Event-locked classification

The results for the event-locked classification of the different error potentials can be seen in Table 1. For execution vs. outcome error, the mean accuracy over all subjects varied between 70.6 and 75.5% depending on the features that were used. While the classification for outcome error against noError reached mean accuracies between 73.9 and 75.6%, the classification of execution errors against noError trials was significantly worse (*p* < 0.05, Wilcoxon's ranksum test) with accuracies between 64.2 and 66.0%.

**Table 1:** **Classification accuracies based on EEG data obtained by 10-fold cross-validation**.

**Subject**	**Execution vs. Outcome**			**Outcome vs. noError**			**Execution vs. noError**		
	**T(%)**	**F(%)**	**T+F(%)**	**T(%)**	**F(%)**	**T+F(%)**	**T(%)**	**F(%)**	**T+F(%)**
S01	77.9	69.7	78.7	74.4	75.1	75.1	69.7	68.8	69.6
S02	76.3	73.8	75.7	78.5	66.1	78.5	65.4	62.6	66.4
S03	69.6	66.5	69.6	68.2	79.3	68.2	59.9	59.0	60.1
S04	72.1	62.7	72.7	75.0	63.4	74.4	60.1	60.8	60.7
S05	70.2	65.8	73.7	60.3	68.0	60.2	64.3	61.6	68.9
S06	67.7	65.4	67.7	76.5	76.0	76.5	63.4	65.2	63.4
S07	73.6	80.7	72.9	76.3	83.5	76.3	62.8	63.3	62.0
S08	85.0	73.2	85.4	80.4	86.4	80.4	68.6	67.6	71.6
S09	78.1	69.8	77.3	71.3	76.3	72.1	64.5	66.5	65.0
S10	82.1	78.2	81.5	78.0	81.9	78.8	71.2	66. 7	71.8
Mean	75.3	70.6	75.5	73.9	75.6	74.1	65.0	64.2	66.0

*The second line denotes the used features (T: time-domain, F: frequency spectrum). Classes were balanced and all results are significantly above chance level (*p* < 0.05)*.

When comparing the use of different features, the combination of time- and frequency domain features gives overall the best results, but the difference compared to either time- or frequency domain features is not significant (*p* > 0.05).

For each classification result, we performed a permutation test (1200 permutations) to assess significance and found that all of the results presented in Table 1 are significantly above chance level (*p* < 0.05).

To check if the classification might be related to eye or finger movement, we also repeated the classification process on EOG data and the position data of the thumbstick. For execution errors and noError trials, we achieved an average accuracy of 50.9% based on EOG and 52.5% based on the thumbstick position. For outcome error against noError trials, average accuracies of 54.9% (EOG) and 56.0% (thumbstick) could be reached. For the classification of the two error types, execution error and outcome error, we obtained average accuracies of 56.4% (EOG) and 55.3% (thumbstick). For the majority of the subjects, the results were not significantly above chance level.

To answer the question if the severity of an error can be detected based on EEG recordings, we tested different degrees of the execution error against each other by using a cross-validation. Since the angle of the movement was randomly modified with different degrees, we also tested if execution errors with a different degree can be classified, e.g., 45° against 180°. Classification results were around chance level (average accuracies between 47.1 and 50.5%), which is why they are not shown in detail in this paper.

### 3.3. Asynchronous classification

Due to the highly imbalanced nature of this asynchronous classification task, with much more time segments being correct than containing an error, we did not use classification accuracy for performance evaluation, but used a different method. We defined windows containing an error as positive and windows without error as negative. Thereby, a true positive (TP) is a window which was correctly classified as containing an error, while a false negative (FN) is a window that contains an error, but was not classified as such. As a performance measure, we calculated the sensitivity given by the number of TP divided by the total number of windows containing an error, and we calculated the specificity given by true negatives (TN) divided by the total number of windows containing no error. To obtain a performance measure that is independent of the threshold, we calculated sensitivity and specificity for different thresholds ranging from 0 to 1 in 0.01 steps and used the area under the curve (AUC) for performance evaluation. Since AUC is a rather abstract performance measure that makes it hard to catch a glimpse of how well the classification would work in an application centered scenario, we calculated the positive seconds rate for a threshold of 0.8, denoted by PSR_0.8_. This value gives the percentage of seconds, in which an error is present and an error was classified. NSR_0.8_ denotes the negative seconds rate for a threshold of 0.8, which gives the percentage of seconds in which no error has happened and in which no error was classified.

**Figure 5:**
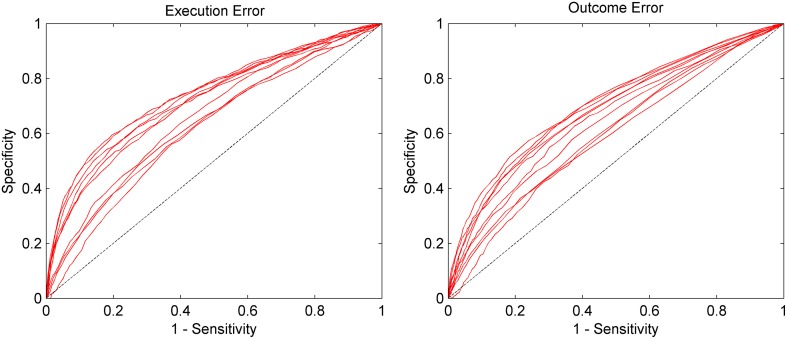
**AUC for the asynchronous classification separated by execution error and outcome error**. Sensitivity (true positive rate) and specificity (true negative rate) were calculated based on the continuous classification in 62.5 ms steps. Each red line represents the data of one subject. The dashed line represents chance level. Results are significantly above chance level for all subjects (*p* < 0.05).

The AUC for the asynchronous classification of the two errors is shown in Figure 5. On average, the AUC for execution error is 0.692, while for the outcome error we obtained an average AUC of 0.657. More detailed results for all subjects can be found in Table 2. The AUC is significantly above chance level for all subjects (*p* < 0.05, permutation test).

**Table 2:** **Classification performance for the asynchronous classification of execution error and outcome error**.

**Subject**	**S01**	**S02**	**S03**	**S04**	**S05**	**S06**	**S07**	**S08**	**S09**	**S10**	**Mean**
**EXECUTION ERROR**											
AUC	0.747	0.745	0.705	0.627	0.723	0.617	0.635	0.664	0.716	0.738	0.692
PSR_0.8_	60.4%	54.8%	49.1%	15.1%	49.9%	44.6%	15.7%	35.5%	58.1%	58.4%	44.2%
NSR_0.8_	70.4%	77.6%	72.5%	89.9%	75.8%	61.2%	90.6%	75.3%	65.9%	71.4%	75.1%
**OUTCOME ERROR**											
AUC	0.700	0.684	0.655	0.593	0.715	0.593	0.609	0.639	0.684	0.701	0.657
PSR_0.8_	17.9%	6.5%	19.7%	0.4%	12.4%	3.1%	0.0%	4.1%	13.4%	9.9%	8.7%
NSR_0.8_	93.6%	97.4%	89.4%	100.0%	98.2%	93.1%	99.9%	97.6%	95.9%	95.8%	96.1%

*AUC denotes the area under the curve for the continuous classification in 62.5 ms steps. PSR_0.8_ denotes the positive seconds rate at a threshold of 0.8, which is the percentages of seconds that contain an error and in which an error was correctly detected. NSR_0.8_ denotes the negative seconds rate at a threshold of 0.8, which is the percentages of seconds that do not contain an error and in which no error was detected*.

## 4. Discussion and conclusion

In this study, we looked at error-related potentials during a video game task with continuous feedback and could show that the two different kinds of error that appeared in this task also produced distinct ErrPs that differ in waveform, latency of its components, and its spectral content. Based on these differences we could use machine learning methods to detect those errors and discriminate between them.

### 4.1. Execution ErrP/interaction ErrP

The execution ErrP found in this study showed four peaks, with the negative peak at 287 ms likely being a FRN, the Pe appearing at around 367 ms and an N400 with a maximum deflection at 486 ms. The shape of the execution ErrP and its topographical distribution is very similar to the typical interaction ErrP described from BCI studies with discrete feedback, in which the user received erroneous feedback from the BCI (Ferrez and del R Millan, 2008; Kreilinger et al., 2012; Spüler et al., 2012a). Comparing the execution ErrP from this study with the results from Krigolson and Holroyd (2007a), who investigated the error-related response during a continuous tracking task, there are notable differences in ErrP waveforms. Krigolson and Holroyd (2007a) introduced errors in which the interface was not responding and found a FRN at 248 ms and a Pe at around 450 ms. While it was shown that the latencies of the ErrP components are task-dependent and that the task can also influence the amplitude of the N400 (Iturrate et al., 2013), there is also evidence that the appearance of the first positive peak and the N400 is not visible in all BCI tasks (Spüler et al., 2012b). Therefore, the differences in waveform and latencies of the potential can likely be explained by the differences in the tasks between the studies.

In the presented study, we additionally analyzed the spectral properties of the EEG signal and found an error-related spectral response, mainly in the delta and theta band. This frequency range is similar to the low frequency component identified by Milekovic et al. (2012) in ECoG recordings and similar to the frequency range used by Omedes et al. (2014).

#### 4.1.1. Influence of error severity

Although we investigated possible effects of the severity of an execution error on the ErrP, we did not find any significant effects and were not able to detect the severity of an execution error (e.g., deflection by 45° or 180°) based on the EEG. This is in contrast to earlier works by Bernstein et al. (1995) and Falkenstein et al. (1996), who showed that the amplitude of the ErrP depends on the difference between the expected and the actual feedback. That we could not find an effect of the severity might be explained by our task design and that the error might be perceived as equally severe by the subject, although the degree of deflection is greater in the 180° condition than in the 45° condition. In a future study, it might be worth investigating different smaller degrees of error (e.g., 15°, 30°, and 45°) and to make sure that these different error degrees are also perceived as differently severe by the subjects.

### 4.2. Outcome ErrP

When looking at the topographic distribution of execution and outcome error, both seem similar with a maximum around electrode FCz and Cz, which indicates that the activity might originate from anterior cingulate cortex (ACC). However, the shape of both ErrPs is very different. The outcome ErrP shows a broad negativity around 2 ms after feedback. That the ERN appears so early, can be explained by the subjects recognizing that a collision is going to happen in advance of the collision actually happening. This is in line with the results by Krigolson and Holroyd (2007b) who have found that predictive feedback leads to an earlier latency of the ERN.

The latency of the Pe for outcome errors at around 264 ms is also about 100 ms earlier than the Pe during execution errors. In contrast, the N400 appears about the same time in both errors, but has a stronger and broader deflection in the outcome error. 742 ms after the error has happened, there is also a small positive deflection.

Since the outcome error shows in general a longer response (from 0 to 750 ms after error), and has lower-frequency peaks than the execution error (happening 200–500 ms after error), these difference enabled us to use machine learning methods to discriminate both errors in time-domain and spectral-domain.

### 4.3. Classification

For classification of the ErrPs, we could show that in an event-locked classification a window of 200 ms to 900 ms gave the best results and execution errors could be detected with an average accuracy around 65%, while the classification of outcome errors achieved an average accuracy around 75%. Also, the two different types of error could be discriminated well with an average accuracy around 75%. Regarding the choice of features, there was no significant difference if temporal and/or spectral features were used for classification.

In the case of an asynchronous classification, the results were different. Although the results were not shown in detail in this publication, the use of only spectral features yielded much higher classification performance in the asynchronous case, which is not astounding, since the data is not event-locked anymore (which is important for time-domain classification). Also, we found that a much shorter window of 100 ms to 500 ms gave optimal results for an asynchronous classification.

However, the overall classification accuracies obtained in this study are lower than in studies using non-continuous BCIs (c.f. Spüler et al., 2012a). As an established method was used for the time-locked classification and the results presented here are lower than in studies using the same method (Spüler et al., 2012a), the classification method itself can be ruled out as a reason for the discrepancy. Since (Holroyd et al., 2009) found the amplitude of the FRN to depend on the degree of which the outcome is perceived to be influenced by the subjects' behavior, one could argue that the lacking influence of the subject on the execution errors is the reason for a weaker ErrP and thereby lower classification accuracies. However, this explanation is less likely since in BCI studies the majority of errors are made by the BCI system and thereby out of the subjects' control. While the simple fact that the difference between continuous and non-continuous feedback could lead to lower classification accuracies would be one explanation, we think the main reason for the lower classification accuracy is the task complexity. Compared to using a BCI, the video game task in this study is rather complex and will likely lead to higher workload than the use of standard BCI systems. Since workload was found to negatively correlate with ERP amplitude (Allison and Polich, 2008), this could be one explanation for the lower classification accuracy, but the relationship between workload and ErrP amplitude, respectively classification accuracy, still needs to be clarified.

### 4.4. Implications for BCI

At last, it needs to be discussed what implications this study has for current BCI research. Since the observed execution ErrP is similar to the ErrPs observed in BCI applications, we expect that results from this task can be transferred to BCI applications and that this data^1^ can be used to improve methods toward ErrP detection in continuous BCIs. For synchronous BCIs giving discrete feedback, ErrPs have been utilized for error correction (Spüler et al., 2012a) and adaptation (Spüler et al., 2012b). By showing that ErrPs can be detected asynchronously during continuous feedback, ErrP-based correction and adaptation can be used for asynchronous EEG-based BCIs and be used to improve existing adaptive decoding methods (Gürel and Mehring, 2012). The fact that execution and outcome errors can be discriminated allows to combine adaptation and error-correcting mechanisms in one BCI system. If an execution error is detected, this information can be used for adaptation of the system, while the detection of an outcome error can be used for error correction. The asynchronous classification also gives a first estimate what accuracies can be expected. This is important information for the design of ErrP-based adaptation algorithms for continuous BCI systems, since the amount of uncertainty in the ErrP detection is a crucial factor that influences the reliability of the adaptation.

### 4.5. Conclusion

Regarding the three questions we mentioned in the introduction and that we wanted to answer with this study, we can conclude the following: (i) ErrPs can be measured in EEG during a cursor control task with continuous feedback, as well as a spectral error-related response (mainly in delta and theta band). They further can be classified in an event-locked, as well as in an asynchronous manner. (ii) The different kinds of errors show a different potential in the EEG with different latency and characteristic of the ErrP components, as well as a different spectral response, which allows a discrimination between execution and outcome errors. (iii) We did not find any significant effect regarding the severity of an error and therefore could not detect it.

## Author contributions

MS conceived and designed the study, performed the analysis, supervised the work and wrote the paper. CN collected the data, performed the analysis and contributed to writing the paper.

### Conflict of interest statement

The authors declare that the research was conducted in the absence of any commercial or financial relationships that could be construed as a potential conflict of interest.

## Footnotes

^1^The data will be made publicly available on the website of the first author.
